# Removing registration holds reduced alcohol prevention program completion without improving college student retention

**DOI:** 10.3389/fpubh.2026.1830979

**Published:** 2026-06-29

**Authors:** Avery Turner, Ashley N. Linden-Carmichael, Jimmy Howard, Kristin Perry, Alexis Drakatos, Leslie D. Leve

**Affiliations:** 1 Prevention Science Institute, University of Oregon, Eugene, OR, United States; 2 College of Education, University of Oregon, Eugene, OR, United States; 3 Division of Student Life, University of Oregon, Eugene, OR, United States

**Keywords:** alcohol, college, digital intervention, equity, online, prevention, university

## Abstract

**Introduction:**

Several online alcohol prevention programs have been developed to reduce alcohol use and risky drinking behaviors among university students in the United States (U.S.). Many large universities have adopted these programs due to their high fidelity and low staff burden, though little research to date has investigated the implementation of these programs.

**Method:**

The current study leveraged a program implementation change at a large public university to investigate the effect of registration holds on program completion rates and student retention rates at the university. Registration holds were used to prevent students from continuing their enrollment at the university if they did not complete the required online alcohol prevention program within their first term of enrollment. The university stopped using registration holds to enforce program completion after concerns were raised that they were inhibiting student retention at the university.

**Results:**

This program evaluation indicated that when the university stopped using registration holds to promote completion of an online alcohol prevention program, program completion rates decreased from 87.9–98.6% in prior years to 81.2%, while student retention at the university did not change. An analysis of historical program data demonstrated that students who engaged in frequent alcohol use, students over the age of 18, male students, and Black and American Indian or Alaskan Native students were less likely to complete an optional follow-up program component.

**Discussion:**

Findings suggest that registration holds improved program completion rates and were not associated with decreased university student retention rates. Lower program completion rates may result in some students benefiting from prevention programming while others are left behind. College administrators and alcohol researchers working with college populations may use these findings to guide implementation decisions and spur future research about factors that influence the implementation of online alcohol prevention programs at colleges and universities.

## 1 Introduction

Alcohol use is highly prevalent among college students; data from the 2024 National Survey on Drug Use and Health indicate that 43.6% of full-time college students report past-month alcohol use, 24.2% report binge drinking in the past month, and 11.4% meet the criteria for a past-year alcohol use disorder (1). Heavy alcohol use contributes to suicide attempts, sexual violence, academic problems, and other negative consequences among college students in the United States (U.S.) (2). The first 6 weeks of a student’s college career is known as a high-risk period for alcohol misuse as students adjust to newfound freedom and new schedules, and are exposed to new peer groups, social pressures, and expectancies related to drinking in the college context (2). The majority of incoming first-year students at public 4-year colleges in the U.S. are 18 years old (3), marking the beginning of emerging adulthood, the developmental period associated with the highest rate of heavy alcohol use across the lifespan (4). Preventing heavy alcohol use and negative alcohol-related consequences is imperative for improving well-being during college and also for reducing the risk of heavy drinking later in life; a longitudinal study found that students who drank heavily during college were more likely to report symptoms of alcohol use disorder at age 35, indicating that heavy drinking during college may have lasting impacts on drinking behavior and health throughout and beyond emerging adulthood (5).

The seriousness of alcohol-related harms and the ubiquity of alcohol use in U.S. colleges make prevention of alcohol misuse imperative. As such, the Safe Drug-Free School and Communities Act of 1989 requires all colleges that receive federal funding to offer alcohol prevention resources to students (6). In order to comply with the Drug-Free Schools and Communities Act of 1989, many colleges require their students to complete an alcohol prevention program during or just prior to their first term of enrollment, which are increasingly delivered online (7). The online format allows for personalization, self-pacing, improved intervention fidelity, and lower staff burden than in-person alternatives (8).

Two widely-used online alcohol prevention programs for college students, Alcohol-Wise and AlcoholEDU, have been found to be effective for reducing alcohol use frequency and heavy episodic drinking in the short-term (9–12). Large-scale evaluations of these programs have drawn their samples from predominantly White institutions and race-specific efficacy analyses were not conducted (9, 12). This lack of subgroup efficacy data is important because alcohol-related risks and consequences are not distributed equally across student populations. Prior research suggests that students identifying as Black and Native American may be at increased risk of alcohol-related harms (13–15). Sex differences further shape alcohol-related risks in college; although female students use alcohol at a slightly higher rate than male students, male students still binge drink at a higher rate (1). Students’ alcohol use also tends to change as they age, with older college students drinking more frequently, but in lower quantities (16). As such, these demographic differences are important for understanding how students engage with alcohol prevention programs even though further research is needed to determine if these demographic features moderate program effectiveness. Consequently, it remains unclear whether lower participation among certain student groups translates into unequal access to program benefits.

Both Alcohol-Wise and AlcoholEDU contain two parts: part 1 takes approximately 1–2 h to complete and part 2 takes 15–30 min. Part 1 for both courses assesses the student’s current alcohol use and related behaviors, guides students through educational modules about safe drinking practices, and concludes with a knowledge test to ensure that students have understood the content. Thirty days after completing part 1 of either program, students are invited to complete part 2. This brief follow-up reviews educational content covered in part 1, which may boost the effectiveness of the program and prolong program effects (17). Part 2 also asks students to again report on their current alcohol use and related behaviors, allowing institutions to compare pre-to-post program alcohol use as a measure of program effectiveness. In addition to their highly similar structure, both Alcohol-Wise and AlcoholEDU cover similar educational material (e.g., protective behavioral strategies, how to recognize signs of alcohol overdose, resources for seeking help, etc.) and provide students with personalized feedback about their drinking behaviors. While these programs are highly similar in structure and content, AlcoholEDU is more widely used and considered more effective than Alcohol-Wise (18).

Effectiveness evaluations of online alcohol prevention programs have noted the importance of the implementation strategy used; Taylor et al. (8) found that only 35% of students at a public university in the Midwest completed both parts of AlcoholEDU when the second part was not required. The racial and gender makeup of the students who completed both parts of the program differed significantly from the full student body. This is important because program effectiveness is often measured by comparing students’ responses on parts 1 and 2; when relatively few students complete the follow-up component, the resulting effectiveness data may underrepresent certain student demographics. A multi-site randomized control trial that aimed to evaluate the effectiveness of AlcoholEDU found that all the colleges that enforced program completion with registration holds achieved at least a 70% completion rate, compared to an average 36% completion rate among colleges that told students the program was required but did not penalize them for not completing it (12). Although the study reported the implementation strategies used by the included colleges and their corresponding completion rates, the primary aims of their study did not include an evaluation of the implementation strategies employed by each college. Prior research suggests that when program completion is not enforced, fewer students complete the prevention program, limiting the reach of the intervention and potentially leaving universities with unrepresentative data about students’ alcohol use and the program’s effectiveness.

While several studies report descriptive or correlational information on strategies used to implement online alcohol prevention programs in college settings, the authors did not identify any peer-reviewed studies that explicitly aimed to compare program completion rates across more than one implementation strategy for online alcohol prevention programs in college populations. Additionally, the authors were unable to find a review of the implementation strategies that are currently used by colleges to implement these programs, so we are unable to summarize the strategies that are used nor each strategy’s relative popularity. Considerable attention has been paid to the effectiveness of online alcohol prevention programs in recent years as their popularity has increased (7). However, now that the effectiveness of these programs is well-established for at least changing short-term behaviors, researchers and college administrators must shift their focus to the strategies that are used to implement these programs. Without a thorough investigation of the implementation strategies used, effective programs may be under-utilized or may disproportionately benefit certain populations of students more than others.

To fill this gap, we examined the impact of one implementation strategy—registration holds—on completion of a required online alcohol prevention program. Registration holds promoted participation by preventing students who did not complete the program from registering for their next academic term. Although this strategy may increase program completion, it may also decrease university student retention by creating an additional barrier to course registration. In addition, because an optional program component (part 2) delivered follow-up prevention content, examining which students completed this component provided insight into whether certain student groups were less likely to engage with prevention programming when participation was not enforced. Using program data from a university that first implemented its online alcohol prevention program with registration holds and later removed them, we aimed to: (1) compare program completion rates before and after the removal of registration holds, (2) compare student retention rates before and after the removal of registration holds, and (3) identify demographic differences between students who completed Alcohol-Wise’s optional follow-up survey and those who did not. This natural experiment provided a rare opportunity to evaluate the effects of an implementation strategy within a single university context, reducing institutional variation that has limited prior comparisons of alcohol prevention program implementation. Findings from this study may inform how U.S. colleges implement and evaluate online alcohol prevention programs.

## 2 Materials and methods

### 2.1 Study context

Prior to Fall 2024, a large public university in the Pacific Northwest required that all first-year students complete Alcohol-Wise within their first 7 weeks of enrollment. This university follows the quarter system, with fall term typically starting in late September, winter term beginning in early January, spring term beginning in late March, and summer term beginning in late June. Students may enroll in any term, but most start in the fall. The university enforced the Alcohol-Wise requirement by placing registration holds on all students who did not complete the program by the specified deadline, thereby making it impossible for students who did not complete the program to maintain their enrollment. The Alcohol-Wise follow-up survey was optional and made available to students 30 days after they completed the required portion of the program (part 1).

Beginning in Fall 2024, the university discontinued their use of registration holds to enforce program completion and switched to AlcoholEDU. The removal of registration holds was prompted by concerns that registration holds negatively impacted student retention at the university, particularly for historically underrepresented student groups. The change in program was spurred by student-reported technical difficulties with Alcohol-Wise and the greater effectiveness rating of AlcoholEDU by the National Institute on Alcoholism and Alcohol Abuse (18). Both programs use a similar structure and delivery mode, suggesting that any observed changes in completion rates are more likely attributable to the removal of registration holds than to differences between the programs themselves. Although no punitive actions were taken against students who did not complete part 1, it was still communicated to students that completing part 1 of the program was required and part 2 was still presented as optional. Additionally, students were sent weekly emails reminding them to complete Alcohol-Wise prior to Fall 2024, though only two reminders to complete AlcoholEDU were sent in Fall 2024.

### 2.2 Study procedures

In part 1 of both programs, students provided demographic information about themselves, along with self-reported alcohol use and related behaviors (see Table 1). These data and the aggregate program completion rates were obtained from the makers of each program. Student retention data were obtained from the university’s Office of Institutional Research. Retention data were captured at the end of the fourth week of fall term. Because some students who initially enroll at the university withdraw prior to the fall census, the retention cohorts are smaller than the total number of students enrolled in the prevention program each academic year. Additionally, retention was only measured from fall term to winter term, whereas the total program enrollment numbers include first-year students who began in winter and spring term. This retention period was selected because the vast majority of students (96.3%) complete the online alcohol prevention program in fall term, so the largest number of holds were placed at the end of fall term and prevented students from continuing their enrollment to their first winter term. Data for Aims 1 and 3 were self-reported on the online alcohol prevention programs whereas data for Aim 2 (student retention analysis) were collected by the university.

**Table 1:** Baseline student demographics by academic year.

Variable	2019–2020 *N* = 6,307	2020–2021 *N* = 5,157	2021–2022 *N* = 6,037	2022–2023 *N* = 6,279	2023–2024 *N* = 6,140	2024–2025 *N* = 3,915
Age						
Under 18	233 (4%)	217 (4%)	230 (4%)	302 (5%)	265 (4%)	86 (2%)
18	3,778 (65%)	3,238 (64%)	3,828 (65%)	4,453 (71%)	4,253 (69%)	2,867 (75%)
19	776 (13%)	708 (14%)	841 (14%)	754 (12%)	799 (13%)	808 (21%)
20+	1,038 (18%)	930 (18%)	1,016 (17%)	769 (12%)	823 (13%)	66 (2%)
Sex						
Male	2,568 (44%)	2,235 (44%)	2,583 (44%)	2,698 (43%)	2,642 (43%)	609 (42%)
Female	3,257 (56%)	2,858 (56%)	3,332 (56%)	3,363 (54%)	3,320 (54%)	843 (58%)
Non-binary	Not provided as an option			217 (4%)	178 (3%)	0 (0%)
Lives on campus	4,303 (74%)	3,087 (61%)	4,346 (73%)	5,141 (82%)	4,898 (80%)	3,646 (95%)
Current alcohol use	3,018 (52%)	2,414 (47%)	2,735 (46%)	3,058 (49%)	2,846 (46%)	2,128 (58%)
Any binge drinking	1,416 (43%)	1,122 (42%)	1,291 (43%)	1,391 (42%)	1,224 (40%)	763 (53%)
Race						
White	4,460 (71%)	3,980 (77%)	4,649 (77%)	Not collected		2,841 (76%)
Black or African American	300 (5%)	289 (6%)	345 (6%)			217 (6%)
Asian	875 (14%)	682 (13%)	809 (13%)			540 (15%)
Hispanic or Latino	798 (13%)	725 (14%)	857 (14%)			689 (19%)
American Indian or Alaska Native	142 (2%)	91 (2%)	127 (2%)			100 (3%)
Native Hawaiian or Pacific Islander	138 (2%)	117 (2%)	133 (2%)			64 (2%)
Middle Eastern/North African	Not provided as an option					71 (2%)
Other	147 (2%)	142 (3%)	154 (3%)			13 (0.3%)

Student-level data from 2018 to 2019 could not be obtained, so it is not included here. Alcohol use was measured by asking how often the student has a drink containing alcohol between academic years beginning in 2019–2023 (any response other than never was coded as “yes”) whereas in 2024–2025 students were asked if they had drank in the past year. Binge drinking was measured by asking students how many times in the past 60 days they consumed 5 + drinks in a row between academic years beginning in 2019–2023, whereas in 2024–2025 students were asked how often in the past 2 weeks they had consumed 4 + drinks (for female students) or 5 + drinks (for male students) within a 2 h period. In both cases, any response greater than 0 was coded as “yes.” Students were able to select multiple race categories so the percentages for all categories sum to greater than 100%. The total Ns presented for each year represent the number of students in each year who provided any demographic data.

### 2.3 Aim 1: program completion rates before and after registration hold removal

#### 2.3.1 Sample

All students who enrolled in the university for the first time between Fall 2018–Spring 2025 were included in this analysis (*n* = 42,970). Because demographic information could only be collected from students who provided this information through the program, Table 1 does not contain data from students who were enrolled in the program but did not provide any demographic data (either because they skipped those questions or did not participate in the program at all). Student-level data could not be obtained from Alcohol-Wise in academic year 2018–2019, so student demographics from that year are not included in Table 1. The 2018–2019 cohort was still included in this analysis because completion rates were available for that year.

Students who qualified for an exemption from completing the program were removed from the count of total enrollees. Beginning in Spring 2022, students could request an exemption from the program by emailing the Office of the Dean of Students and stating that they identified as a student in recovery, a student parent, were over the age of 25, or worked full time. Students who were exempted from the program were sent a shorter online module to complete that was designed specifically for students in recovery and/or non-traditional students. Instructions for requesting an exemption were included in the initial program invitation email. Students who reported no alcohol use were not exempt from the program given that these universal prevention programs are designed to be helpful for non-drinkers (e.g., teaching students how to recognize signs of an overdose among their peers) and normalize abstinence by sharing with students the proportion of their peers who report not drinking. It is critical for universities to collect data from these students to better understand how many students are not drinking and their reasons for abstaining, which are variables that are collected via the program. Additionally, the educational content is useful for students who report not drinking on part 1 of the program but who start using alcohol later in their college career.

#### 2.3.2 Measures

##### 2.3.2.1 Grouping variable: academic year

All academic years with registration holds (academic years beginning in 2018–2023) were compared to the academic year without holds (2024**–**2025).

##### 2.3.2.2 Outcome: part 1 completion

Students were counted as having completed the program if they clicked through all the educational materials and passed the final knowledge exam at a rate of 70% for Alcohol-Wise or 80% for AlcoholEDU. The passing rate for Alcohol-Wise was set by the maker of the program, whereas the university selected the pass rate for AlcoholEDU.

#### 2.3.3 Analytic approach

A series of chi-square tests were used to determine if the rate of part 1 completion in 2024–2025 (when no registration holds were used) was significantly lower than the rate of part 1 completion in each prior year (academic years beginning in 2018–2023). A Holm correction was applied to adjust for multiple comparisons. No missingness was observed in this analysis because students either completed the program or did not.

### 2.4 Aim 2: student retention rates before and after registration hold removal

#### 2.4.1 Sample

A total of 32,634 first-time students were counted in the week four of fall term census across 2018–2024. Of those, more than half were female (18,643; 57.1%). Most were 18 years old (26,829; 82.2%) and were enrolled full-time (32,182; 98.6%). A total of 7,430 (22.8%) were first-generation college students and 7,868 (24.1%) were eligible for a Pell grant. About two-thirds identified as White (60.2%), 16.5% as Hispanic or Latino, 9.2% as two or more races, 7.0% as Asian, 3.0% as Black/African American, 1.5% as international students, 0.5% as American Indian or Alaskan Native, and 0.5% as Native Hawaiian or Other Pacific Islander. An additional 526 students’ race was unknown because they did not report their race on their application for admission.

#### 2.4.2 Measures

##### 2.4.2.1 Predictors: demographic variables

Demographic variables used in the retention analyses were collected via the student’s application for admission. Students self-reported their sex assigned at birth (male or female; non-binary was not included in the university-provided retention data), age, if they were a first-generation college student (yes/no), if they were eligible for a Pell grant (yes/no), and were prompted to select one or more of the following race categories: American Indian or Alaskan Native, Asian, Black or African American, Hispanic or Latino, Native Hawaiian or Other Pacific Islander, White, or International student. When students selected more than one race, they were placed into a “two or more races” category unless they selected international student, in which case they were only counted as an international student. As such, these racial categories are mutually exclusive.

Small cell sizes in the post-registration hold removal condition, which only included academic year 2024–2025, necessitated re-categorizing race. The small count of students identifying as American Indian or Alaskan Native and Native Hawaiian or Other Pacific Islander warranted the creation of a Black, Indigenous, and People of Color (BIPOC) category. This category combined students who identified as Black or African American, Hispanic or Latino, American Indian or Alaskan Native and Native Hawaiian or Other Pacific Islander. The decision to combine these races, as opposed to just the races with small sample sizes, was driven by the documented lower retention and graduation rates of BIPOC students relative to White and Asian students at predominantly White-serving institutions (19). International students were excluded from this analysis due to their small sample size in Fall 2024 and our inability to reasonably combine them with any other group. Students whose race or ethnicity was unknown were excluded because differential retention rates for students with an unknown race would not have provided useful data for the institution to consider in terms of program implementation. Additionally, we were unable to compare retention rates across full-time and part-time students, given the small number of part-time students in the Fall 2024 cohort (*n* = 49). Both full- and part-time students were included in analyses.

##### 2.4.2.2 Outcome: student retention

Students who enrolled for the first time in fall term between 2018 and 2024 were considered retained when they maintained their enrollment at the university through the fourth week of their first winter term. This retention period was selected because most holds were placed in fall term and prevented winter term registration.

#### 2.4.3 Analytic approach

Two-sample proportion tests were used to compare retention rates between academic years beginning in 2018–2023 (when registration holds were in place) to the academic year beginning in 2024 (no registration holds). The overall student retention rate was compared over these two time periods, as well as the retention rates for each level of sex, age, race, first generation status, and Pell eligibility. A Holm correction was applied to adjust for multiple comparisons. No missingness was observed in this analysis because the university tracks enrollment for all students.

### 2.5 Aim 3: student demographics of optional program component completers

#### 2.5.1 Sample

Because student-level data could only be obtained from Alcohol-Wise after Fall 2019 and race was not collected beyond Spring 2022, this sample was restricted to academic years 2019–2020, 2020–2021, and 2021–2022. Students who did not complete either part of the program (*n* = 724) were excluded from this analysis because the purpose of this aim was to identify demographic differences between students who completed only the required program component and those who additionally completed the optional follow-up component. A total of 16,777 students completed part 1 of the program during these academic years. This is slightly higher than the total number of part 1 completers in Table 2 because some students completed the program even though they were later granted an exemption, which was likely pursued to avoid additional institutional requirements to complete the online cannabis and other drugs prevention programs which were also implemented with registration holds (program data for these additional modules are planned but were not included in the current report). In total, the three required online drug prevention programs (alcohol, cannabis, and other drugs) are estimated to take students up to 3 h to complete.

**Table 2:** Number of program completers by part per year.

Academic year	Total enrolled in program	Part 1 completers	Part 2 completers
2018–2019	6,461	5,679	3,872
2019–2020	6,308	5,788	3,431
2020–2021	5,156	5,086	2,563
2021–2022	5,938	5,795	3,652
2022–2023	6,491	6,250	3,523
2023–2024	6,337	6,047	2,643
2024–2025	6,279	5,101	404

Students who qualified for an exemption from the program were removed from these counts.

#### 2.5.2 Measures

##### 2.5.2.1 Predictors: demographic variables

In part 1 of Alcohol-Wise, students self-reported their age, sex assigned at birth, and their race. Students who were 18 years old and male students were treated as the reference groups for age and sex, respectively. Students could select multiple races from the options presented: White, Black or African American, Asian, Hispanic or Latino, American Indian or Alaskan Native, Native Hawaiian or Pacific Islander, or Other. Seven binary race variables were created to indicate that a student identified as that race (coded 1) or did not (coded 0). As such, the reference group for each race category was students who did not self-identify as that race. Finally, students were asked “How often do you have a drink containing alcohol?” and responded with one of the following options: “Never or N/A. I don’t drink alcohol.”, “Monthly or less than monthly”, “Two to four times a month”, “Two to three times a week”, or “Four or more times a week.” Never drinking was the reference group for alcohol use frequency. The week in the term that students started part 1 of the program was added to the model to adjust for differences in exposure to the university setting. The week in the term variable was coded such that 1 indicated the first week of the term, while 0 and negative numbers indicated that the student began the program before the term began. Additionally, a categorical academic year variable was added to the model to adjust for cohort and history effects, which were particularly relevant given the onset of the COVID-19 pandemic during this period.

##### 2.5.2.2 Outcome: part 2 completion

Any student who opened the link to part 2 was considered a part 2 completer, given that they may have reviewed the educational materials without providing any data. This generous measure of part 2 participation also allows for the inclusion of students who opted not to provide personal information at the follow-up but still were willing to engage with this optional program component.

#### 2.5.3 Analytic approach

A single logistic regression model was fit to the data predicting follow-up completion (coded 0 or 1). Predictors included all levels of age, sex, drinking frequency, and all seven binary race variables. Because binary race variables were used, students who selected more than one race were counted across more than one race variable. While this is not ideal for modeling, it more accurately represents the student’s identities and gives the results more clear implications (i.e., significantly lower part 2 completion for students who are “two or more races” does not provide clear policy guidance). Additionally, pulling students into a “two or more races” category would disproportionately affect smaller racial categories. However, such an approach may have better captured the experiences of multiracial students compared to disaggregating their identities across multiple race categories. Multiracial students face unique barriers to college enrollment and retention compared to monoracial students (20), indicating that their experiences may not be adequately reflected when their identities are parsed across multiple race variables. On the other hand, dummy coding race variables did not require selecting a reference group, whereas mutually exclusive categories would have forced all results to be relative to one race category. In Aim 2, small cell sizes necessitated the combination of multiple race categories to create a BIPOC race category, but small cell sizes were not a barrier in this analysis because data from 3 academic years were analyzed together. Although consistency in our operationalization of race throughout this study would be ideal, differences in the way that race was collected by the university (mutually exclusive categories used in the retention analyses) and the prevention programs (not mutually exclusive) required flexibility in our approach.

Because demographic features were self-reported in part 1 and completion of part 1 was inclusion criteria for this analysis, only 15 cases were missing data on any of these demographic variables. There was no missingness on the outcome (part 2 completion). Given that less than 1% of the data were missing, listwise deletion was used.

## 3 Results

### 3.1 Aim 1: program completion rates before and after registration hold removal

**Figure 1:**
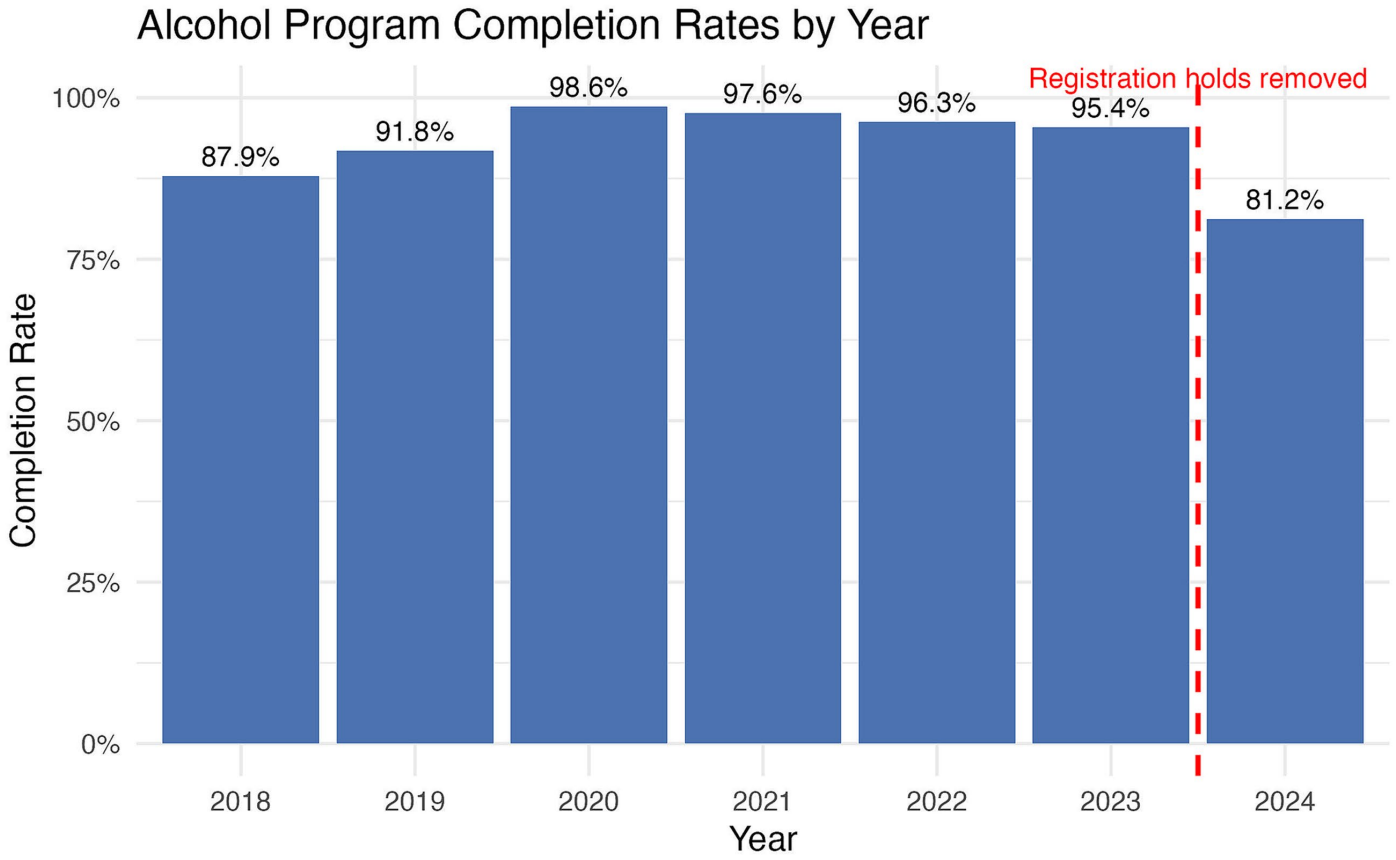
Online alcohol prevention program part 1 completion rate by year.

In the first year without registration holds (academic year 2024–2025), 81.2% of students completed the required portion of the online alcohol prevention program (part 1), which is the lowest rate in the time period observed (prior year rates ranged from 87.9–98.6%; see Figure 1 and Table 2). A series of chi-square tests with a Holm correction revealed that the part 1 completion rate in 2024–2025 was significantly lower than all prior years (adjusted *p*s < 0.01). Cohen’s *h* effect sizes ranged from 0.19–0.66. A sharp decline in part 2 completions in 2024–2025 was also observed, with only 6.4% of enrolled students completing both parts of AlcoholEDU. About half of students had completed both parts of Alcohol-Wise in academic years beginning 2019–2023 (range = 41.7–61.5% completion rate).

### 3.2 Aim 2: student retention rates before and after registration hold removal

Two-sample proportion tests with Holm correction showed no significant change in student retention when overall retention rates were compared pre-2024 to rates in academic year 2024–2025 (see Table 3). No significant changes in student retention were detected for any of the tested demographic subgroups of students (each level of sex, age, race/ethnicity, first generation status, and Pell eligibility) when retention rates for these groups were compared pre-2024 to rates in academic year 2024–2025.

**Table 3:** Students retained from their first term to their second term.

Student characteristic	Registration holds in use (2018–2023)	No registration holds (2024)
Overall	26,459 (96.1%)	4,907 (96.5%)
Sex		
Female	15,101 (96.0%)	2,811 (96.4%)
Male	11,358 (96.1%)	2,096 (96.6%)
Age		
<18	1,134 (95.1%)	171 (95.5%)
18	21,771 (96.2%)	4,056 (96.7%)
19	3,324 (95.8%)	630 (95.5%)
20+	217 (91.2%)	50 (94.3%)
Race/ethnicity		
BIPOC	5,127 (94.0%)	1,175 (94.8%)
Asian	1,874 (97.9%)	360 (98.6%)
White	16,142 (96.5%)	2,819 (96.8%)
Two or more races	2,412 (95.8%)	471 (96.9%)
First generation status		
Yes	5,862 (93.3%)	1,074 (93.9%)
No	20,597 (96.9%)	3,833 (97.2%)
Pell eligibility		
Eligible	6,063 (93.7%)	1,315 (94.0%)
Not Eligible	20,392 (96.8%)	3,592 (97.4%)

BIPOC combined students who identified as Black or African American, Hispanic or Latino, American Indian or Alaskan Native and Native Hawaiian or Other Pacific Islander.

### 3.3 Aim 3: demographic differences in students who opt to complete an optional program component and those who do not

After adjusting for all other demographic characteristics and academic year, several demographic characteristics were associated with completion of the optional follow-up component (part 2; see Tables 4, 5). Alcohol use frequency was significantly associated with follow-up completion. Compared with students who reported never drinking, students who reported drinking monthly or less were more likely to complete part 2. In contrast, students who reported drinking two to four times per month and two to three times per week were less likely to complete part 2 than students who reported never drinking. Race was also associated with follow-up completion. White and Asian students were more likely to complete part 2 than students who did not identify as those races. In contrast, Black students and American Indian or Alaska Native students were less likely to complete part 2 than students who did not identify as those races. Hispanic, Native Hawaiian or Pacific Islander, and Other race indicators were not significantly associated with Part 2 completion. Age and sex were also significant predictors. Students aged 19 or older were less likely to complete part 2 than students aged 18, and female students were more likely to complete Part 2 than male students. Students who began part 1 later in the term were less likely to complete part 2. Cohort-specific trends were also observed, with higher follow-up completion in 2021–2022 and lower completion in 2020–2021 compared with 2019–2020.

**Table 4:** Student characteristics by program parts completed.

Student characteristic	Part 1 only *N* = 7,070	Part 1 + Part 2 *N* = 9,707
Week in term prevention program was started	−2.19 (2.19)	−2.73 (1.21)
Age		
Under 18	232 (3.3%)	444 (4.6%)
18	4,126 (58%)	6,705 (69%)
19	1,092 (15%)	1,211 (12%)
20+	1,612 (23%)	1,341 (14%)
Sex		
Female	3,448 (49%)	5,970 (62%)
Male	3,614 (51%)	3,731 (38%)
Race (not mutually exclusive)		
White	5,331 (75%)	7,707 (79%)
Asian	896 (13.0%)	1,467 (15%)
Black or African American	494 (7.0%)	431 (4.4%)
Hispanic or Latino	1,078 (15%)	1,286 (13%)
American Indian or Alaska Native	177 (2.5%)	180 (1.9%)
Native Hawaiian or Pacific Islander	179 (2.5%)	207 (2.1%)
Other Race	376 (5.3%)	426 (4.4%)
Baseline alcohol use		
Never or N/A, I do not drink alcohol	3,603 (51%)	5,032 (52%)
Monthly or less than monthly	1,697 (24%)	2,758 (28%)
Two to four times a month	1,206 (17%)	1,442 (15%)
Two to three times a week	490 (6.9%)	418 (4.3%)
Four or more times a week	69 (1.0%)	53 (0.5%)

Week in the term was computed such that students who began the program during the first week of the academic term were coded as 1. Students who began the program prior to the start of the term were assigned values of 0 or negative integers corresponding to the number of weeks before Week 1 that the program was initiated. Race variables were dummy-coded, so multiracial students are represented in multiple categories.

**Table 5:** Student demographics predicting part 2 completion logistic regression results.

Predictor	OR	95% CI	*p*
Intercept	0.68	[0.59, 0.78]	<0.001
Week in term prevention program was started	0.85	[0.83, 0.87]	<0.001
Baseline alcohol use frequency (ref: never)			
Monthly or less than monthly	1.10	[1.02, 1.18]	0.018
2–4 times a month	0.85	[0.77, 0.93]	<0.001
2–3 times a week	0.69	[0.60, 0.80]	<0.001
4+ times a week	0.79	[0.54, 1.15]	0.219
Age (ref: 18)			
<18	1.16	[0.98, 1.37]	0.085
19	0.78	[0.71, 0.86]	<0.001
20+	0.69	[0.63, 0.76]	<0.001
Race (not mutually exclusive)			
White	1.27	[1.15, 1.41]	<0.001
Asian	1.34	[1.20, 1.50]	<0.001
Black or African American	0.70	[0.60, 0.80]	<0.001
Hispanic or Latino	0.93	[0.83, 1.03]	0.146
American Indian or Alaskan Native	0.74	[0.60, 0.92]	0.007
Native Hawaiian or Pacific Islander	0.88	[0.65, 1.18]	0.387
Other race	0.99	[0.80, 1.22]	0.894
Sex (ref: male)			
Female	1.58	[1.49, 1.69]	<0.001
Academic year (ref: 2019–2020)			
2020–2021	0.72	[0.67, 0.78]	<0.001
2021–2022	1.18	[1.10, 1.28]	<0.001

Race variables were dummy-coded so odds ratios should be interpreted as odds of completing part 2 for a student who selects that race compared to students who did not select that race.

## 4 Discussion

While online alcohol prevention programs are effective for reducing alcohol use among college students, there has been little investigation into how these programs are implemented and the downstream effects of specific implementation strategies. The goal of this program evaluation was to examine the effect of using registration holds to promote participation in a required online alcohol prevention program on program completion and university student retention. Additionally, we examined which demographic groups were more likely to complete optional program components to shed light on student groups that might be missed when participation is not enforced. Results suggest that registration holds increase completion of online alcohol prevention programs without reducing student retention. Students who completed the optional follow-up portion of the program differed demographically from those who completed only the required portion, indicating that follow-up program data may not be representative of the broader student population. Notably, students historically underrepresented in higher education—including Black and American Indian or Alaskan Native students and students older than 18—were less likely to complete optional program components when completion was not enforced. This suggests that when registration holds are removed, lower completion rates may partly reflect decreased participation among historically underrepresented students relative to White or Asian students and students aged 18. These findings are consistent with prior research showing that students participate at higher rates when registration holds are used (12) and that students who complete optional program components may not be representative of the student body (8). Unlike prior research, the current study examined implementation strategies within a single university and directly tested differences in completion rates across strategies. By doing so, the study reduced between-university variation that has limited prior research and allowed for statistical comparisons of completion rates across implementation strategies.

Although academic year 2024–2025 had the lowest completion rate of any year tested, the part 1 completion rate remained much higher than the part 2 completion rate in prior years. This discrepancy may be the result of the university describing part 1 as “required” even after registration holds were removed, whereas the part 2 follow-up has always been presented to students as optional. Additionally, the lower part 2 completion rate in 2024–2025 relative to prior years indicates that there may be a downstream effect of the lower part 1 completion rate, which is problematic because program effectiveness is often measured by comparing self-reported alcohol use on parts 1 and 2. Registration holds may operate to improve program completion by increasing the perceived cost of non-completion and signaling to students that alcohol prevention is an institutional priority. Student engagement with the program may also be different under registration holds; research on mandated behavioral health treatment suggests that perceived coercion may influence participant engagement and satisfaction, even when mandated participation does not necessarily result in poorer outcomes overall (21). Substance use prevention programming differs substantially from substance use disorder treatment, however, students’ perceived lack of choice in participating under registration holds likely influenced their willingness to engage with the program.

The finding that retention rates did not improve nor worsen after registration holds were removed implies that registration holds may have relatively little cost to students in terms of their ability to maintain their enrollment, though receiving a registration hold may be stressful for students and burdensome for staff to administer. The Western Interstate Commission for Higher Education conducted focus groups with 50 students from 10 institutions to understand how students navigated registration holds (22). Students reported that receiving a hold made them feel as though their institution did not care about them and they wished that the communication from their institution regarding the hold was clearer. An analysis of administrative data at a large public university indicated that students who had high financial need, identified as Black or African American, and were first-generation college students were more likely to receive a registration hold in their first 2 years of enrollment than students who did not have financial need, identified as another race, and were not the first in their family to attend college (23). These findings indicate that receiving a hold may be disheartening and confusing for students, and that the inequitable application of holds could contribute to existing barriers to college enrollment for historically underrepresented college students. In both studies, registration holds for financial reasons (e.g., past-due balance) were included so the effect of the hold may have been compounded by the student’s financial stress and be substantively different from receiving a hold for other reasons, such as not completing a required online alcohol prevention program. Our results suggest that student retention did not change after registration holds were removed, but the effects of receiving a registration hold on student mental well-being and mental health was not measured in this analysis.

Although prior research suggests that registration holds have been applied inequitably, their removal in this context may have resulted in inequities in participation in alcohol prevention programming. The finding that students who drank 2–4 times per month and 2–3 times per week were less likely to complete the optional follow-up program component (part 2) than students who do not use alcohol indicates that as the program completion rate declines, students who are drinking frequently may be missed because they are opting out of participation. Equally concerning are the lower part 2 completion rates observed among American Indian or Alaskan Native and Black or African American students relative to students of other races. Interventions for alcohol use may be particularly impactful for these populations because American Indians have the highest rates of alcohol use of any racial group (15) and racial discrimination against Black college students has been linked to increased alcohol use (14). National trends show slightly higher rates of alcohol use among female college students, though males still outpace females in binge drinking and heavy alcohol use (1), making the lower completion rates for male students concerning. These findings indicate that the students who might benefit most from alcohol prevention programs may be missed when the program completion rate declines.

### 4.1 Limitations

This natural experimental design had the strength of eliminating between-college variation, although this also reduced the generalizability of the findings. However, the online alcohol prevention programs that were implemented are widely used among large public universities in the U. S., increasing the utility of these findings. The change in implementation strategy was confounded with the change in program in the current study, but we believe that the impact of this is minimal because the delivery mode and format of both programs are very similar. However, the reduction in reminder emails may also have contributed to lower completion rates. The lack of change in retention rates may have been driven by the high retention rate while registration holds were in use, resulting in a ceiling effect.

While multiple academic years under registration holds were included, we only analyzed 1 year post-registration hold removal. It may take several years to observe changes in student behavior after a policy change is implemented; in a review of smoke and tobacco-free policies at universities in the U.S., many studies had a follow-up period of multiple years after the policy change and noted changes over that time such as increases in acceptability of the policy in the 4 years following implementation (24). Relatedly, de-implementing programs that are harmful or not effective takes time, evidenced by the continued use of the Drug Abuse Resistance Education (D.A.R.E.) program decades after it was found to be ineffective for preventing youth substance use (25). Thus, the one-year follow-up period after registration holds were removed may not be sufficient for observing the full effects of this change on program completion rates and student retention.

The current study was further limited by the availability of data. Alcohol-Wise was unable to provide the university with student-level data for academic year 2018. Additionally, we did not have any demographic data about students who did not participate in the program at all so we could not estimate the likelihood of completing part 1 based on student demographics. Student retention data were only provided to the research team in aggregate form and therefore could not be linked to individual student data from the programs. This limited our ability to investigate the impact of heavy drinking on continued enrollment and how many students completed the program but were not retained (i.e., students who left the university for other reasons). Relatedly, all program data were de-identified, which precluded us from analyzing which students received a registration hold.

### 4.2 Future directions

Although it was a strength of this study that two implementation strategies were tested within the same university setting, replication across multiple universities would provide insight into whether the effects of removing registration holds are context specific. The current study did not explore indicators of program engagement beyond completion, which may be relevant for determining whether students are engaging differently when they face penalties for not completing the program (i.e., students may be clicking through content without meaningfully engaging when they are just trying to avoid a registration hold). Future research should also cover a longer post-registration hold removal period to assess the long-term impacts on program completion rates and student retention rates. Additionally, a punitive strategy was implemented and removed in this study, but incentive-based strategies for promoting program engagement should also be explored. Finally, the authors were unable to find a review paper that summarized the implementation strategies currently in use by universities to implement online alcohol prevention programs, which would be helpful for understanding the universe of implementation strategies that exist and their relative popularity.

## 5 Conclusion

This program evaluation provides support for the use of registration holds to promote completion of online alcohol prevention programs for college students. We found that discontinuing registration holds—used to enforce completion of a required online alcohol prevention program by preventing students who did not complete the program from registering for their next academic term—did not increase student retention rates. Additionally, students who completed optional program components differed demographically from those who did not, indicating that program effectiveness data derived from follow-up surveys may not reflect the broader student population. While more research is needed to establish best practices for implementing online alcohol prevention programs, this study provides information that may help college administrators and alcohol researchers make informed decisions about how these programs are implemented in U.S. college settings.

## Data Availability

The data analyzed in this study is subject to the following licenses/restrictions: due to institutional data use agreements and student privacy protections, the data are not publicly available. De-identified analytic data may be made available upon request and with appropriate institutional approval. Requests to access these datasets should be directed to leve@uoregon.edu.
